# Ultralight shape-recovering plate mechanical metamaterials

**DOI:** 10.1038/ncomms10019

**Published:** 2015-12-03

**Authors:** Keivan Davami, Lin Zhao, Eric Lu, John Cortes, Chen Lin, Drew E. Lilley, Prashant K. Purohit, Igor Bargatin

**Affiliations:** 1 Department of Mechanical Engineering and Applied Mechanics, University of Pennsylvania, Philadelphia, Pennsylvania 19104, USA; 2 Nanotechology Master's Program, University of Pennsylvania, Philadelphia, Pennsylvania 19104, USA; 3 Vagelos Integrated Program in Energy Research, University of Pennsylvania, Philadelphia, Pennsylvania 19104, USA

## Abstract

Unusual mechanical properties of mechanical metamaterials are determined by their carefully designed and tightly controlled geometry at the macro- or nanoscale. We introduce a class of nanoscale mechanical metamaterials created by forming continuous corrugated plates out of ultrathin films. Using a periodic three-dimensional architecture characteristic of mechanical metamaterials, we fabricate free-standing plates up to 2 cm in size out of aluminium oxide films as thin as 25 nm. The plates are formed by atomic layer deposition of ultrathin alumina films on a lithographically patterned silicon wafer, followed by complete removal of the silicon substrate. Unlike unpatterned ultrathin films, which tend to warp or even roll up because of residual stress gradients, our plate metamaterials can be engineered to be extremely flat. They weigh as little as 0.1 g cm^−2^ and have the ability to ‘pop-back' to their original shape without damage even after undergoing multiple sharp bends of more than 90°.

## Abstract (web summary)

Unusual mechanical properties can be obtained with careful design of metamaterials. Here, Davami *et al*. use a microscale periodic cellular design to create plate mechanical metamaterials which exhibit ultralow mass per unit area, enhanced bending stiffness and ability to recover after extreme deformations.

## Graphical abstract

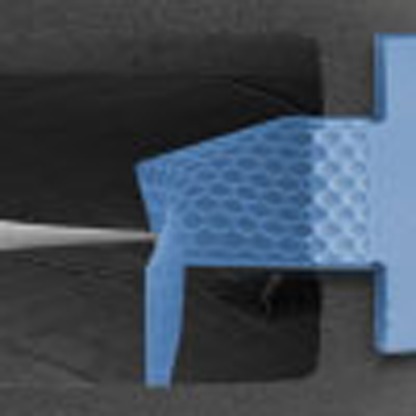

Advances in micro/nanofabrication techniques recently resulted in demonstrations of macroscopic solids made solely out of free-standing films with nanoscale thickness^1^. These mechanical metamaterials have a carefully engineered structure at the micro- and nanoscales, with periodic geometries that lead to unusual mechanical properties. For example, mechanical metamaterials can be engineered to have a record high stiffness for a given effective density or have a very large range of elastic deformation^1^,^2^,^3^,^4^,^5^. The examples reported in literature typically have a truss-like structure with the underlying material forming an interconnected periodic framework that is easily penetrated by air or other ambient. Such ‘bulk' metamaterials can be fabricated to occupy a macroscopic volume in all three dimensions^2^, subject only to the limitations of the used fabrication method. However, they cannot by themselves be formed into a continuous plate that could serve, for example, as the wing of a flying microrobot.

Mechanical metamaterials are only the most recent development in the ongoing quest for materials with high stiffness and low density. In fact, they can be viewed as a sub-class of cellular materials^6^, which have long been used in many applications, ranging from thermal insulation^7^ to aerospace structural elements^8^. Aerogel materials are some of the oldest and most celebrated cellular materials due to their ultralow weight and thermal conductivity. Since their discovery in the 1930s (ref. 9), much effort has been dedicated to tailoring aerogels' properties for various applications. Aerogels with ultralow densities (1 mg cm^−3^ or less) have been fabricated from silica, alumina and carbon^10^,^11^,^12^,^13^; however, such aerogels are typically very brittle, with tensile, compressive and shear strengths in the order of 1 MPa or less^14^, which significantly limits their applications^15^. More recently, a number of low-density carbon-based materials composed of sheets as thin as a single atomic layer^16^,^17^,^18^,^19^ have been demonstrated. These often have unique mechanical properties; however, as with aerogels, the micro/nanoscale geometry of these cellular materials can only be partly controlled and is largely random.

Typically, the effective Young's modulus, *E*_eff_, of a cellular structure scales as 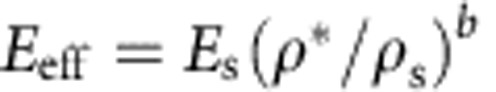, where 
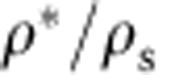
 is the relative density, equal to the ratio of the apparent density of the cellular material to that of the underlying solid film (that is, cell wall) material, *E*_s_, is the Young's modulus of the cell wall material, and the power-law exponent *b* is determined by the dominant deformation mode in the cellular material. In materials with bending-dominated deformation, such as aerogels and foams, there is a quadratic or stronger relationship between the Young's modulus and density, implying that material properties degrade very quickly as the apparent density is reduced^6^. However, it is possible to obtain a lower exponent *b* by carefully engineering the architecture of the constituent elements of the cellular material^20^. Some of the recently reported truss-like metamaterials exhibit the ideal linear relationship (*b*=1) between the stiffness and density^4^, allowing them to occupy new areas^4^,^5^ on the stiffness-density Ashby chart^21^. Other truss-like mechanical metamaterials fabricated out of ultrathin films (<100 nm) featured exponents *b* between 1 and 2, but were surprisingly robust, recovering their original shape after deformations of up to 50% even when manufactured out of brittle materials such as alumina^5^. In contrast to random cellular architecture typical of foams, nanoscale mechanical metamaterials can therefore have superior stiffness and strength at very low densities because the constituent materials are distributed more regularly and efficiently throughout the structure.

In addition to bulk mechanical metamaterials, periodic cellular architectures have been used extensively in the design of stiff and lightweight plates. This is typically achieved by patterning the plate in the normal direction, that is, perpendicular to the plane of the sheet. For example, simple corrugated sheets consisting of a single three-dimensional patterned layer can have significantly enhanced bending stiffness^22^ and have found a wide range of applications from macroscale architecture^23^ to nanoscale sensors^24^ and energy devices^25^. Honeycomb lattices and sandwich structures, which consist of two face sheets attached to a periodic cellular core, have become ubiquitous in construction, aerospace, scientific instrumentation (for example, optical tables) and other industries that require lightweight rigid plates^26^,^27^. Although sandwich structures possess a high bending stiffness, they typically cannot sustain a sharp bend without fracture.

Here, we use microscale periodic cellular design to create robust, stiff, and ultra-lightweight plates out of ultrathin films created by atomic layer deposition (ALD). Similar to truss-like ‘bulk' metamaterials, our plate metamaterials can sustain extreme deformations when made out of ALD films with thicknesses below 100 nm; although truss-like metamaterials can recover after large compression or shear deformations, plate metamaterials can recover from extreme bending deformations. In contrast to truss-like bulk metamaterials, plate metamaterials achieve such robustness while remaining geometrically continuous, that is, without any structural holes. In addition, plate metamaterials can be fabricated in high volumes and with relatively large lateral dimensions using standard optical lithography techniques. While even thinner suspended films have been made out of graphene and other two-dimensional materials^28^,^29^,^30^, the resulting membranes are often prone to tearing, warping or crumpling. As a result, such membranes are typically much less than 1 mm in lateral dimensions^29^,^30^ and remain flat only if put under tension on a rigid frame. In contrast, our free-standing plates are completely standalone and mechanically robust even at truly macroscale lateral dimensions (>1 cm).

## Results

### Fabrication and geometry of honeycomb plate metamaterials

**Figure 1:**
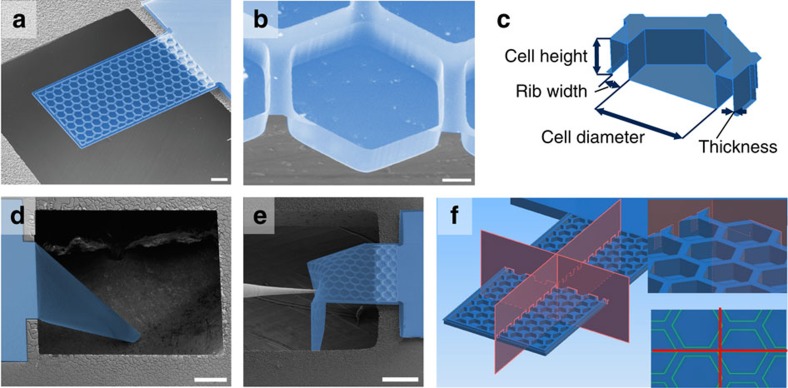
Geometry of honeycomb plate metamaterials. (**a**) An scanning electron microscopic (SEM) image of a 1-mm-long cantilevered plate made using a hexagonal honeycomb metamaterial geometry. The 35-nm-thick ALD alumina film is coloured for clarity. Scale bar, 100 μm. (**b**) An SEM image of the detail of the cell structure. Scale bar, 10 μm. (**c**) A schematic representation showing an individual cell with its characteristic dimensions. (**d**) An SEM image of a 50-nm-thick cantilever without the honeycomb pattern, illustrating the warping typical of suspended flat ultrathin films. Scale bar, 200 μm. (**e**) An SEM image of a 1-mm-long 25-nm-thick plate bent by more than 90° using a micromanipulator without any fracture. After the tip of the micromanipulator was removed, the plate recovered its original shape (see Supplementary Movie 1, Supplementary Note 2 and Supplementary Figs 3 and 4). Scale bar, 200 μm. (**f**) Schematic illustrating the requirement that any plane perpendicular to the plate will intersect vertical walls. The insets show zoomed-in detail of how the plane intersects individual cells.

Figure 1a shows a cantilevered (single-clamped) rectangular plate based on a hexagonal honeycomb metamaterial design. The plates were fabricated out of ALD alumina films with uniform thickness, which varied between 25 and 100 nm in different fabrication runs, using optical lithography and a combination of wet and dry etching techniques (see the Supplementary Note 1, Supplementary Figs 1 and 2, and (ref. 31) for detail of the fabrication process). Alumina material was chosen as the cell wall material for its high stiffness, chemical and high-temperature resistance, as well as the highly conformal and pinhole-free nature of its ALD films.

As shown in Fig. 1b,c, the plate is able to maintain its shape because of its three-dimensional honeycomb microstructure, which increases the flexural stiffness of the plate by one to three orders of magnitude depending on the used cell parameters (see Supplementary Note 3 for detail). When bending stresses are applied, rigidity is provided by the vertical walls and the horizontal segments between the ‘hexagonal cups' and the ‘cup' bottoms similar to the way that C-beams or corrugated sheets resist deformation. This keeps the sheet flat even when the film has significant internal stresses due to fabrication-induced stress gradients that generally cause unpatterned films to curl up with a small radius of curvature (Fig. 1d).

### Robustness of plate metamaterials

In contrast to macroscale honeycomb sandwich structures, our plates can sustain large bending deformations because these top and bottom surfaces are not continuous, allowing the structures to fold with a very small radius of curvature without fracture, as shown in Fig. 1e. Despite the brittleness of the cell wall ceramic material (aluminium oxide), these ultrathin structures are robust and typically recover their original shapes even after undergoing extreme bending deformations multiple times (Supplementary Movie 1). As with previously reported bulk metamaterials^5^, structures made out of thinner films were typically more robust, that is, able to withstand sharper bends. This is simply because of the fact that the maximum strain along the fold lines formed in these plates at large bending deformations is proportional to the thickness of the solid cell wall film. In this aspect, plate metamaterials are very different from plates with effectively uniform cross-sections, such as corrugated sheets or sandwich plates, for which the classical plate theory holds and the maximum strains are therefore proportional to the curvature multiplied by the height of the plate rather than the thickness of the face sheets. If a sandwich plate undergoes a sharp bend (that is, with radius of curvature similar to the plate height), the induced strains are of the order of one, which would lead to failure of most structural materials. This remains true for sandwich plates made of films with macro-, micro- or nanoscale thickness. In contrast, the maximum strains in our plates are determined by the ratio of film thickness to plate height, which is in the order of 10^−3^ for the thinnest ALD plates we fabricated, explaining their observed robustness.

The design of the particular features and dimensions of the plates was guided by the results of COMSOL finite element simulations (see Supplementary Note 3). Based on the results of the simulations, we have focused our experiments on plates with hexagonal honeycomb geometry because of their approximately isotropic bending stiffness for small deformations^6^,^32^. Other periodic geometries are possible, but to achieve high flatness and bending stiffness, it is crucial that any plane perpendicular to the plate must intersect vertical walls (Fig. 1f). Otherwise, the plane will bend very easily along a line that contains only horizontal elements. Among regular polygon tiling patterns, only hexagonal (honeycomb) patterns can satisfy this requirement. Other, lower-symmetry tiling patterns, such as basket-weave or rhombille, also satisfy this requirement, but generally have a lower bending stiffness (see Supplementary Note 3, Supplementary Fig. 7, and Supplementary Table 1).

### Enhanced bending stiffness of plate metamaterials

**Figure 2:**
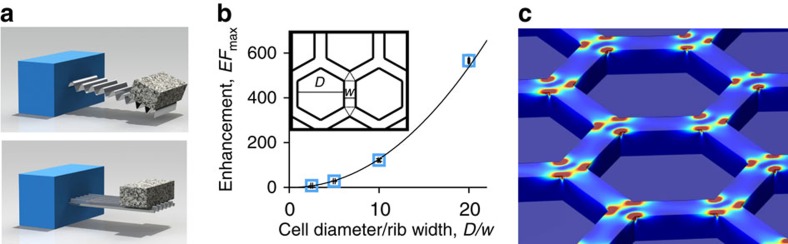
Simulated bending properties of traditional corrugated sheets and plate metamaterials. (**a**) Illustration of the anisotropic bending stiffness of periodic corrugated sheets with a constant cross-section. (**b**) Isotropic enhancement factor of honeycomb corrugated sheets versus the ratio of cell diameter to rib width. (**c**) COMSOL finite element simulation illustrating the concentration of elastic energy in the areas where the ribs intersect. The colour represents the elastic energy density, with red areas corresponding to the highest elastic energy density and blue to the lowest.

Traditional prismatic corrugated sheets with a periodic cross-section (Fig. 2a) offer a greatly enhanced bending stiffness in one direction but almost no change in the bending stiffness in the perpendicular direction^22^,^23^. The enhancement factor, EF, which can be used to describe the increase in bending stiffness relative to a completely planar sheet, is determined by the moment of inertia of its constant cross section. Typically, it scales quadratically with the ratio of height of the corrugation, *h*, to the thickness of the sheet, *t*, and depends only weakly on the other geometric parameters (for example, period) of the corrugation pattern: 
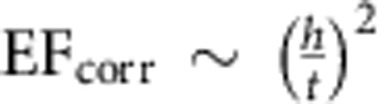
.

In contrast, the hexagonal corrugation used in this work offers an enhanced bending stiffness in all directions, and its isotropic enhancement factor depends on all parameters of the unit cell. We have studied this dependence through extensive finite element simulations of small-deformation response in COMSOL (see Supplementary Note 3 and Supplementary Figs 5 and 6). Briefly, the enhancement factor of ultrathin plates initially increases quickly with height, just like in simple corrugated sheets, but then saturates for heights much greater than the film thickness. This maximum saturated value of the enhancement factor depends on the ratio of two in-plane parameters of the cell geometry: 
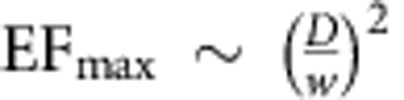
, where *D* is the inner diameter of the hexagons and *w* is the width of the ribs (Fig. 2b).

An elastic energy analysis can be used to explain these results for the maximum enhancement factor, as well as the overall dependence of the bending stiffness on the height of our honeycomb plates (see Supplementary Note 3 for detail). Briefly, for sufficiently large heights, the hexagons with their attached vertical ribs become very rigid, and practically do not bend. As a result, when one of our honeycomb plates is bent, the majority of the elastic energy is concentrated in the areas near the hexagon vertices, where the strengthening ribs intersect. Figure 2c shows one representative case, where ∼95% of the total elastic energy is concentrated in these triangular regions according to COMSOL simulations. Thus, the total area undergoing bending in a honeycomb plate is only a small fraction of the total area, and the bending stiffness is enhanced proportionally to the ratio of these areas.

More specifically, consider a unit cell of area *A* of a honeycomb patterned plate. Let the area occupied by the small triangular regions (near each vertex) per unit cell be *A*_2_ and therefore *A*_2_=*A*−*A*_1_ is the area of the remaining part of the unit cell. The corresponding mean curvature bending moduli of these regions are *K*_1_ and *K*_2_, respectively. If a uniform isotropic moment per unit length *m* is acting on the unit cell, then the elastic energy stored in the patterned plate per unit cell can be written as 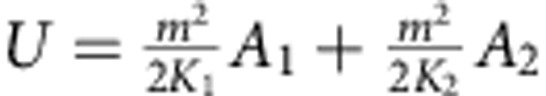. Equating 
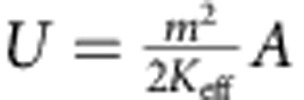
, where *K*_eff_ is the effective (average) bending modulus for the plate metamaterial, we obtain 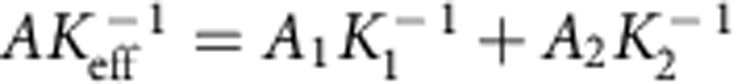. Now, 
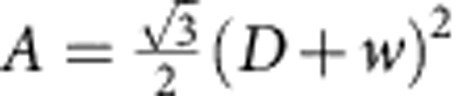
 and 
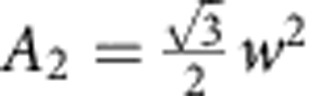
. In the limit of large plate heights, *K*_1_→∞, and the effective bending modulus is given by 
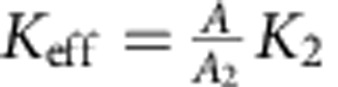
, which gives 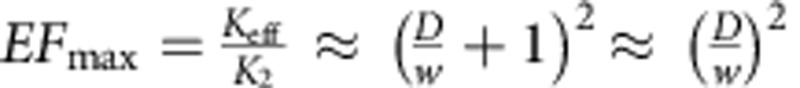
for 
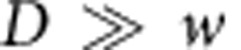
. The latter agrees with the enhancement factor obtained by numerical simulations. More detailed analysis can be found in Supplementary Note 4 and Supplementary Fig. 8.

### Measurements of bending stiffness

To validate the finite element model used for metamaterial designs and determine the Young's modulus of the cell wall ALD alumina material, we have measured the small-deformation bending stiffness of honeycomb plates using an atomic force microscope (AFM). In particular, the spring constants of the cantilevered plates were inferred from the force-displacement measurements obtained using an AFM.

**Figure 3:**
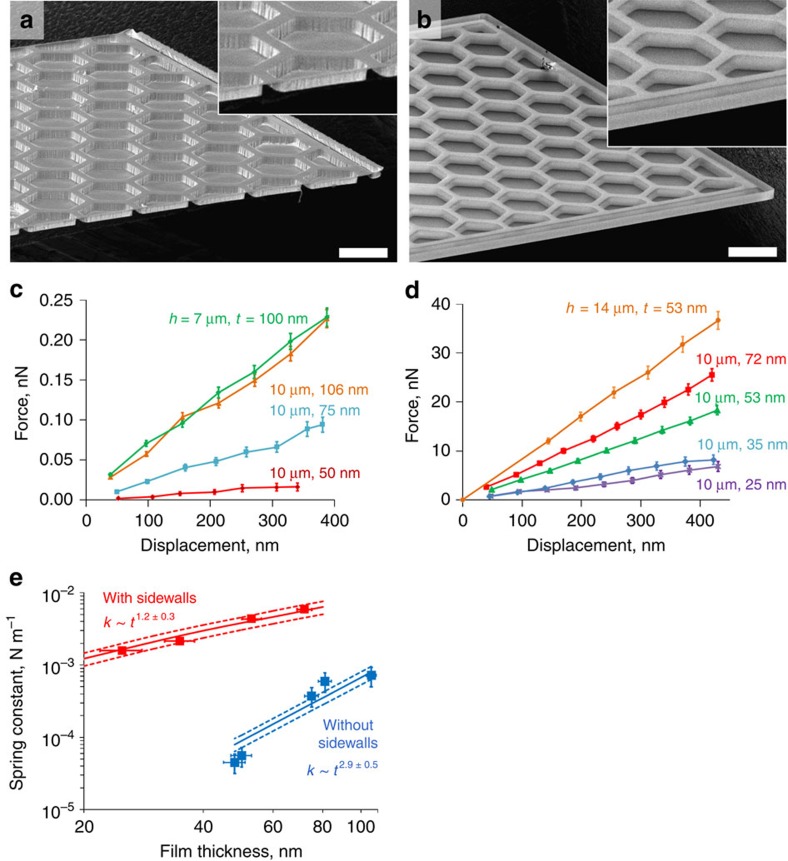
Measured bending response of honeycomb cantilevers with and without sidewalls. Scanning electron micrographs of a 1-mm-long cantilevers (**a**) without and (**b**) with vertical sidewalls, which dominate the spring constant of cantilever when present. Scale bar, 50 μm in both micrographs. The insets show the vertical sidewall detail. (**c**) Force-displacement curves measured for 1-mm-long ALD honeycomb cantilevered plates (without vertical sidewalls) for different thicknesses and heights. Note that cantilevers with a thickness of ∼100 nm and heights of 7 and 10 μm have essentially the same spring constant. (**d**) Same for cantilevers with vertical sidewalls, which increase the overall stiffness of the cantilever by up to two orders of magnitude. Note that the cantilevers with a thickness of ∼50 nm and heights of 10 and 14 μm have dramatically different spring constants, in accordance with the scaling 
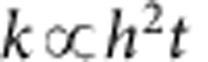
.(**e**) A comparison between experimental results and simulations for honeycomb structures with 10 μm height and different ALD film thicknesses (with and without sidewalls). The curves show the actual predictions of the finite-element simulations (solid lines) along with ±20% confidence interval (dashed lines). The symbols show experimental data. The experimental errors bars denote s.e.m. in **c**,**d** and s.d. in **e**. For the top curve (with sidewalls) in **e**, the vertical error bars are smaller than the symbols.

Somewhat unexpectedly, the measured stiffness of the honeycomb cantilevers depends very strongly on whether continuous vertical walls are present on the sides of the cantilevers (compare Fig. 3a,b). Such walls can be formed at cantilever edges either intentionally by designing them into the lithography mask or unintentionally due to fabrication imperfections. When sidewalls are absent, the cantilever's spring constant 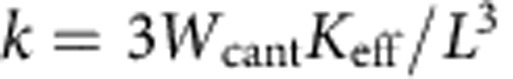 is directly proportional to the bending stiffness of the metamaterial, *K*_eff_, introduced in the previous section, and the cantilever width *W*_cant_. As a result, the spring constant scales cubically with the film thickness, 
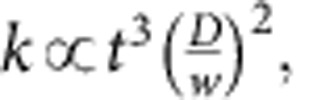
 and is almost independent of the height of the structure (Fig. 3c), as discussed in the previous section. In contrast, when sidewalls are present, the force-displacement response of the cantilevers is dominated by the stiffness of the two C-beams formed by these walls at each side of the cantilever. As a result, the corresponding spring constants scales linearly with thickness and quadratically with plate height, 
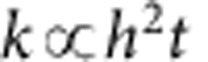
, as expected from the stiffness scaling of conventional C-beams and corrugated sheets (Fig. 3d). Figure 3e compares these experimental results to the spring constants from finite element simulations for plates with a cell height of 10 μm, a cell diameter of 50 μm, and ALD alumina thickness between ∼25 and ∼100 nm. The finite element simulations agree with experimental results best if we assume a Young's modulus of 130 GPa, which is within the range of elastic moduli reported for ALD alumina films in the literature^33^.

To illustrate the repeatability and stability of the elastic behaviour of the ALD honeycomb plates, we also performed cyclic loading tests: a plate with a honeycomb depth of 10 μm and an ALD layer thickness of 72 nm was bent and unloaded 400 times consecutively without exhibiting any significant changes in its spring constant (see Supplementary Note 5 and Supplementary Fig. 9 for detail).

### Properties of centimetre-scale plates

**Figure 4:**
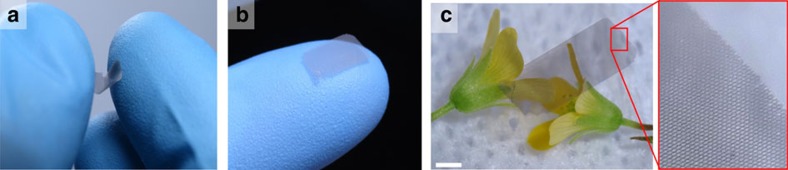
Photographs of large-area honeycomb plates. Before (**a**) and after (**b**) pictures of a bent 0.5 × 1.0 cm honeycomb plate. (**c**) Same plate placed on flower petals does not cause any bending of the petals. Scale bar, 3 mm. The inset zooms in on a section of the plate showing the honeycomb microstructure.

The robustness and flatness of the honeycomb periodic metamaterial allowed us to fabricate honeycomb plates on the macroscopic scale with lateral dimensions on the centimetre scale (Fig. 4). While the ALD layer thickness is only ∼50 nm, the devices do not show any visible warping or deformation. The plates fully recover their shape after bending (Fig. 4a,b). They are also extremely lightweight (Fig. 4c) and float in air, descending very slowly (see Supplementary Movie 2).

For the largest honeycomb plates we made (Fig. 4), the cell parameters shown in Fig. 1c must be simultaneously optimized for high bending stiffness, robustness to defects, and ease of fabrication. In particular, we fixed the minimum feature size at 10 μm to maximize the yield of optical lithography and etching fabrication steps, and that determined the rib width *w*=10 μm in all fabricated samples. While the bending stiffness increases with increasing cell diameter (see Supplementary Note 3), we limited cell diameters to *D*=50 μm in fabricated samples to maximize the robustness of the films. Plates using cells with larger diameters are more prone to damage when probed with a micromanipulator tip or when individual fabrication defects occur because damaging a single cell affects a significant portion of the total structure. In contrast, in our fabricated plates, the fabrication defects or external damage due to, for example, a puncture by an AFM tip, was localized to within a few cells because the cracks typically terminated at the vertical walls (see Supplementary Note 6 and Supplementary Figs 10–12 for detail).

**Figure 5:**
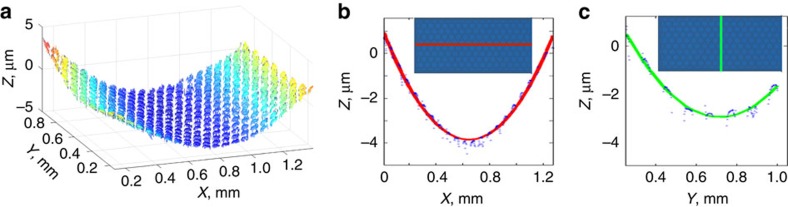
Residual curvature of centimetre-scale plates. (**a**) Profilogram of a free-standing ALD honeycomb plate resting on a flat chuck, obtained using a Zygo white-light optical profilometer. The vertical scale is greatly exaggerated (by approximately two orders of magnitude) to illustrate the small residual curvature of the plate. For this large field of view of the profilometer, the sidewalls and bottom surfaces of the plate are not resolved correctly and therefore do not appear in this plot (see Supplementary Fig. 13 and Supplementary Note 7 for higher-resolution profilograms that show sidewalls and cup bottoms). (**b**) Cross-section profile and the parabolic fit to the top surfaces of the plate, used to determine the radius of curvature of the plate along the *X* direction (*r*_*X*_≈5 cm). The inset shows the location of the cross section. (**c**) Same for the perpendicular *Y* direction (*r*_*Y*_≈11 cm).

The cell height, which can be viewed as the effective thickness of the plate metamaterial, was varied between 1 and 14 μm, corresponding to a range of etching depths easily accessible with standard semiconductor reactive ion etching (RIE) tools. The primary effect of increasing the cell height is an increase in the bending stiffness and flatness (see Supplementary Note 3). The plates with the largest heights of 10−14 μm were remarkably flat, with the typical vertical displacement of only ±4 μm over the 0.8 × 1.2 mm field of view of the profilometer (Fig. 5a). The measured radii of curvature were in the order of 10 cm (Fig. 5b,c).

## Discussion

The mass per unit area (areal density) of our plate metamaterials can be easily determined from the geometry, film thickness and density of the cell wall material. For the typical density of ALD alumina of ∼4 g cm^−3^ (ref. 33), the mass per unit area is between 10 and 40 μg cm^−2^ (0.1−0.4 g m^−2^). For comparison, common soap bubbles and films can be made with thicknesses on the order of 100 nm and therefore areal densities of 0.1 g m^−2^, but they are fluid and ephemeral, unable to maintain their shape due to gravity-induced flow and evaporation^34^. The thinnest large-area plastic sheets that are commonly available are 0.5-μm-thick sheets of Mylar (also known as ‘OS film'), which have an areal density on the order of 0.7 g m^−2^, making them ∼10 times thicker and three times heavier than the ALD plates shown in Fig. 4. At the same time, the flexural stiffness of OS films is approximately twice smaller than that of 50-nm-thick ALD honeycomb plates (see Supplementary Note 8 for detail). As a result, these Mylar films wrinkle easily, sag under their own weight, and generally cannot maintain their shape unless put on a frame under tension (see Supplementary Figs 14 and 15).

The ALD plates we have fabricated have very high aspect ratios: height-to-thickness ratio of up to ∼1,000 and the length-to-height ratio of up to ∼1,000 as well, for an overall length-to-thickness aspect ratio of up to one million. As mentioned earlier, these large height-to-thickness aspect ratios are responsible for the bending robustness of our plates, but such aspect ratios would be very difficult to realize using conventional materials at the macroscale. To illustrate the extreme aspect ratios used to achieve the unique mechanical properties of these structures, it is useful to compare them to origami paper structures, from which some recently reported mechanical metamaterials were made^35^,^36^. If a model of our 50-nm-thick ALD plates (Fig. 4) was made out of standard 100-μm-thick paper (that is, scaled up by a factor of 2,000), the height of the corrugated paper plate would need to be 10 μm × 2,000=20 mm, and the lateral dimensions would be 10 × 20 m—about the size of a standard tennis court. Such large free-standing paper structures obviously cannot maintain their shape under gravity. Only by using cell walls with nanoscale thickness made from a stiff material like alumina we were able to fabricate free-standing plates with such large aspect ratios.

In summary, we have demonstrated shape-recovering, flat, ultrathin, continuous plate metamaterials with enhanced bending stiffness. Similar to the recently reported ‘bulk' mechanical metamaterials^4^, these plates are extremely lightweight and resilient because of their nanoscale thickness and microscale cellular structure, but they also form flat continuous plates. The unusual properties of the plate metamaterials stem from both their hexagonal corrugation geometry, which enhances flatness and bending stiffness, and the extremely low thickness of the film, which reduces weight and increases robustness. They are among the thinnest and lightest free-standing macroscale solids ever made, that is, objects that are chemically stable, able to maintain their shape, and be handled with bare hands.

The continuous nature of the plates, their robustness, and very low weight make them suitable for a number of applications. For example, these plates can be used to make thinner wings than anything created by nature or mankind so far for applications in flying microrobots or microstructures^37^,^38^. The high Young's modulus and the refractory properties of the cell wall alumina material enable such applications even in harsh environments. The extremely low thickness and weight may allow such mechanical metamaterials to serve as acoustic or optical metamaterials as well. Finally, our plate metamaterials also offer a new approach for measuring the mechanical properties of engineered nanoscale materials: Thanks to the large lateral dimensions of the fabricated nanostructured plates, the material properties of these nanoscale materials can be studied at the macroscale using standard test methods and systems, such as Instron materials testing machines.

## Methods

### Fabrication process

The fabrication started with a double side polished Si wafer. SiN films with a thickness of 180 nm were deposited on both sides using plasma-enhanced chemical vapor deposition (PECVD). A hexagonal lattice of regular hexagons (honeycomb structure) with a depth of up to 14 μm were patterned on the front side of the silicon wafer using photolithography and RIE. The back side was patterned via photolithography and RIE etching of SiN to create openings for subsequent release. Next, the SiN mask was removed from the front side using RIE etching and the ALD layer was then deposited using trimethylaluminium and water precursors. The deposition rate was measured using an ellipsometer to be 1.18 Å per cycle at 250 °C. Supplementary Fig. 1a shows a die with all three types of millimetre-scale device fabricated out of plate metamaterial: cantilevers, doubly clamped beams, and rectangular plates clamped on all four sides, while Supplementary Fig. 1b shows one centimetre-scale cantilever on wafer. The schematic of the fabrication procedure is outlined in Supplementary Fig. 1c.

To pattern the ALD layer, a thick layer of SPR 220 resist (MicroChem Corp.) was spin-coated on the structure. After the spin coating and soft baking at 105 °C, the wafer was cooled down slowly to make sure the photoresist did not crack. After photolithography, inductively coupled plasma etching with a BCl_3_-based chemistry process was used to pattern the alumina ALD layer. Next, most of the silicon under the ALD plate was removed using anisotropic KOH etching. Before placing the wafer in KOH, the top surface was covered with ProTEK B3 (Brewer Science Inc.) to prevent the ALD layer from being etched in the KOH solution. Also, since PECVD SiN does not cover the edge of the wafer, a protective chuck was used to keep the edge inaccessible. A silicon etching rate of 75 μm h^−1^ was measured at 80 °C in the 30% KOH solution. By accurately timing the KOH etching process, it was possible to stop the process ∼20 μm from the top surface. The exact depth was measured using a Zygo white-light optical profilometer. After that, the ProTEK layer was removed and a 15-min oxygen plasma cleaning was performed to remove any remaining polymer residue. Isotropic dry XeF_2_ etching was used for the final release of the structure. Approximately 100 cycles (30 s each) of XeF_2_ etching with a ratio of 3.2:2 (XeF_2_:N_2_) was needed to completely release the structures.

### Shape recovery after extreme deformation

To study the flexibility and shape recovery property of the structure, we used a micromanipulator inside a focused ion beam microscope. Honeycomb cantilevers with different ALD layer thicknesses underwent complex loading and deformation. The cantilever recovered its shape after each set of deformations without showing any damage. Structures with thinnest ALD layer films were able to withstand the highest level of deformation repeatedly without damage. Supplementary Fig. 3 illustrates a set of sequential images of a cantilever with a thickness of 25 nm under different loading showing its flexibility and shape recovery. The corresponding video is also available as Supplementary Movie 1.

### Finite element simulations

The small-deformation response of plate metamaterials was simulated using the structural mechanics module of COMSOL Multiphysics (version 5.0). We assumed constant cell wall thickness and isotropic linear elastic properties, with a Young's modulus of 130 GPa and a Poisson ratio of 0.22. Given the extreme aspect ratios of the plate metamaterial, we modelled the structures using thin shell geometry with triangular mesh elements. Geometric nonlinearity was included but did not significantly affect the results for the relatively small deformations used in AFM testing (Fig. 2).

For the simulations of the bending stiffness of an infinite plate (Supplementary Fig. 5), pure bending moments were applied to the loaded ends of a unit cell, and mirror symmetry conditions to the remaining two sides. The bending stiffness from the unit cell simulations was compared to the bending stiffness inferred from the simulated spring constant of cantilevered plates under a point load at the tip (corresponding to AFM measurements), which provided an estimate for the accuracy of finite element simulations.

### AFM measurements

AFM measurements of force-displacement curves were performed using an Asylum atomic force microscope at room temperature and under ambient conditions. Two different types of AFM probes (NANOANDMORE, USA) were used for the characterization. For the honeycomb cantilevers without the vertical sidewalls, the nominal spring constant of the AFM tip was ∼0.01 N m^−1^, and the length, width and thickness of the cantilever were 125 μm, 34 μm and 350 nm, respectively. The measurements on the honeycomb cantilevers with the vertical sidewalls were conducted using an AFM probe with a nominal spring constant of 2 N m^−1^ and a cantilever length of 225 μm, width of 27 μm, and thickness of 2.7 μm. Before the measurement of the ALD plates, the spring constant of the tip, *K*_tip_, was obtained using a thermal noise method.

After calculating the spring constant of the tip as well as obtaining the inverse optical lever sensitivity (InvOLS), a central load was applied at the middle of the free end of the cantilever using the tip. The load was in the range of 0.5–100 nN which is calculated by multiplying the trigger point (0.25 to 0.5 V), InvOLS (∼100 nm V^−1^), and the spring constant of the cantilever (0.01 or ∼2 N m^−1^). The beam displacement can be calculated from *δ*_h_=Δ*Z*_p_−Δ*Z*_c_, where *δ*_h_, Δ*Z*_p_ and Δ*Z*_c_ are the honeycomb cantilever deflection, the AFM piezo travel distance, and the AFM cantilever deflection, respectively. Knowing that 1/*K*_total_=1/*K*_tip_+1/*K*_beam_, and *K*_total_ is the slope of the linear section of the force-displacement graph, the spring constant of the beam can be calculated.

## Additional information

**How to cite this article:** Davami, K. *et al*. Ultralight shape-recovering plate mechanical metamaterials. *Nat. Commun.* 6:10019 doi: 10.1038/ncomms10019 (2015).

## Supplementary Material

Supplementary Information Supplementary Figures 1-15, Supplementary Table 1, Supplementary Notes 1-8 and Supplementary References.

Supplementary Movie 1 1-mm-long cantilevered plates undergoing extreme bending deformation. The plates are deformed using a remotely controlled micromanipulator while imaged using scanning electron microscopy inside a focused ion beam system. The plates with the lowest thickness were the most robust and could fold onto themselves without permanent damage. For comparison, a plate with 1 µm of residual silicon broke at much smaller deformations.

Supplementary Movie 2 Free falls of 1-cm-long plates under gravity. The second clip shows that the plates sometimes floated up due to convection flows that were undetectable using heavier objects. The measuring tape in the background is providing a length scale in inches.
