# Supplementary material for: Ultralight shape-recovering plate mechanical metamaterials
Source: Nat Commun. 2015 Dec 3;6:10019. doi: 10.1038/ncomms10019 (PMC4686658; doi:10.1038/ncomms10019)
Supplement: Supplementary Information — Supplementary Figures 1-15, Supplementary Table 1, Supplementary Notes 1-8 and Supplementary References. [file ncomms10019-s1.pdf]

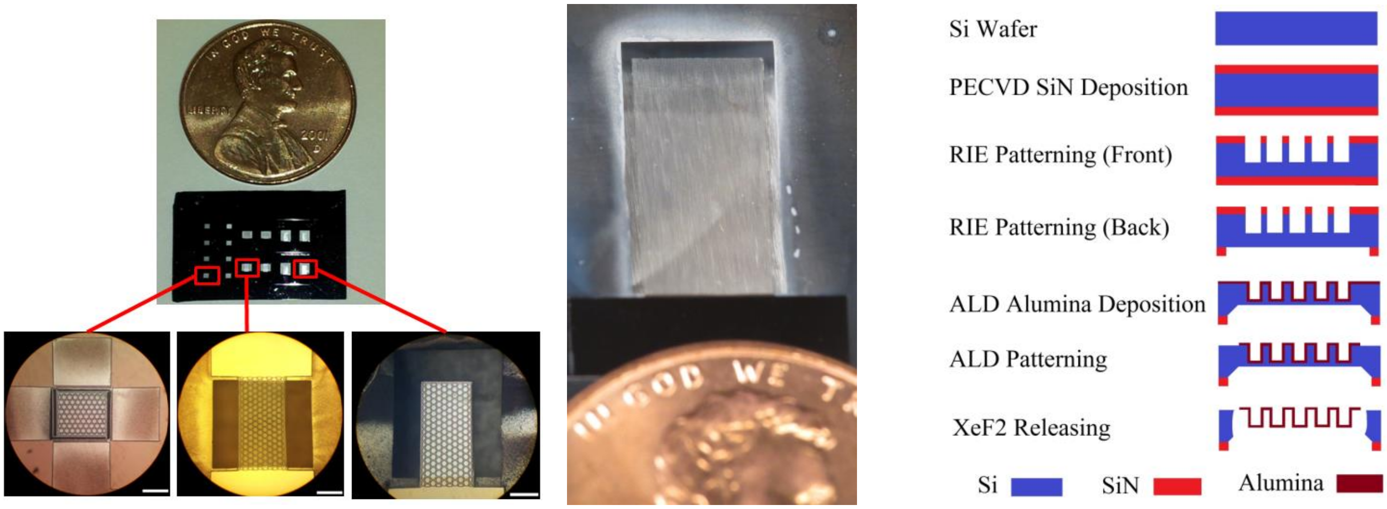

(a) (b) (c)

Supplementary Figure 1: *(a) A finished die next to a US penny for scale (top) and light microscope images (bottom) of the three millimeter-scale device types (cantilever, doubly clamped beam, plate). The scale bar in all the pictures is 250 µm. (b) A centimeter-scale cantilever on a larger die. (c) A schematic of the fabrication process.*

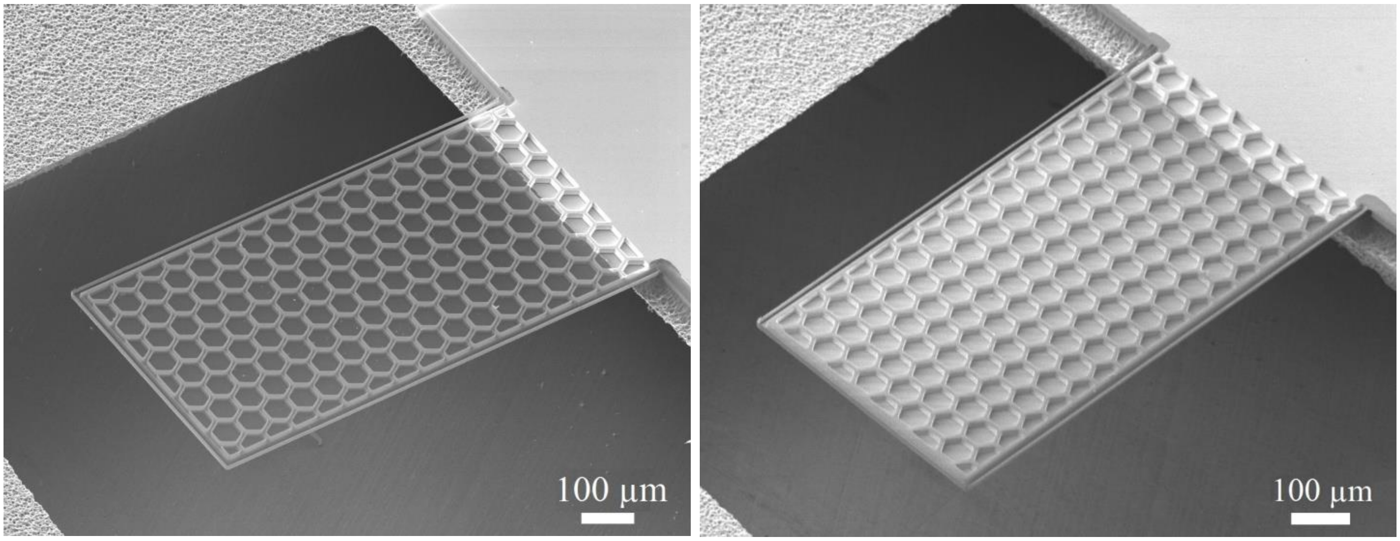

(a) (b)

Supplementary Figure 2: *SEM images of a suspended micro honeycomb structure (a) under a 15.0 kV acceleration voltage, (b) under a 2.0 kV acceleration voltage.*

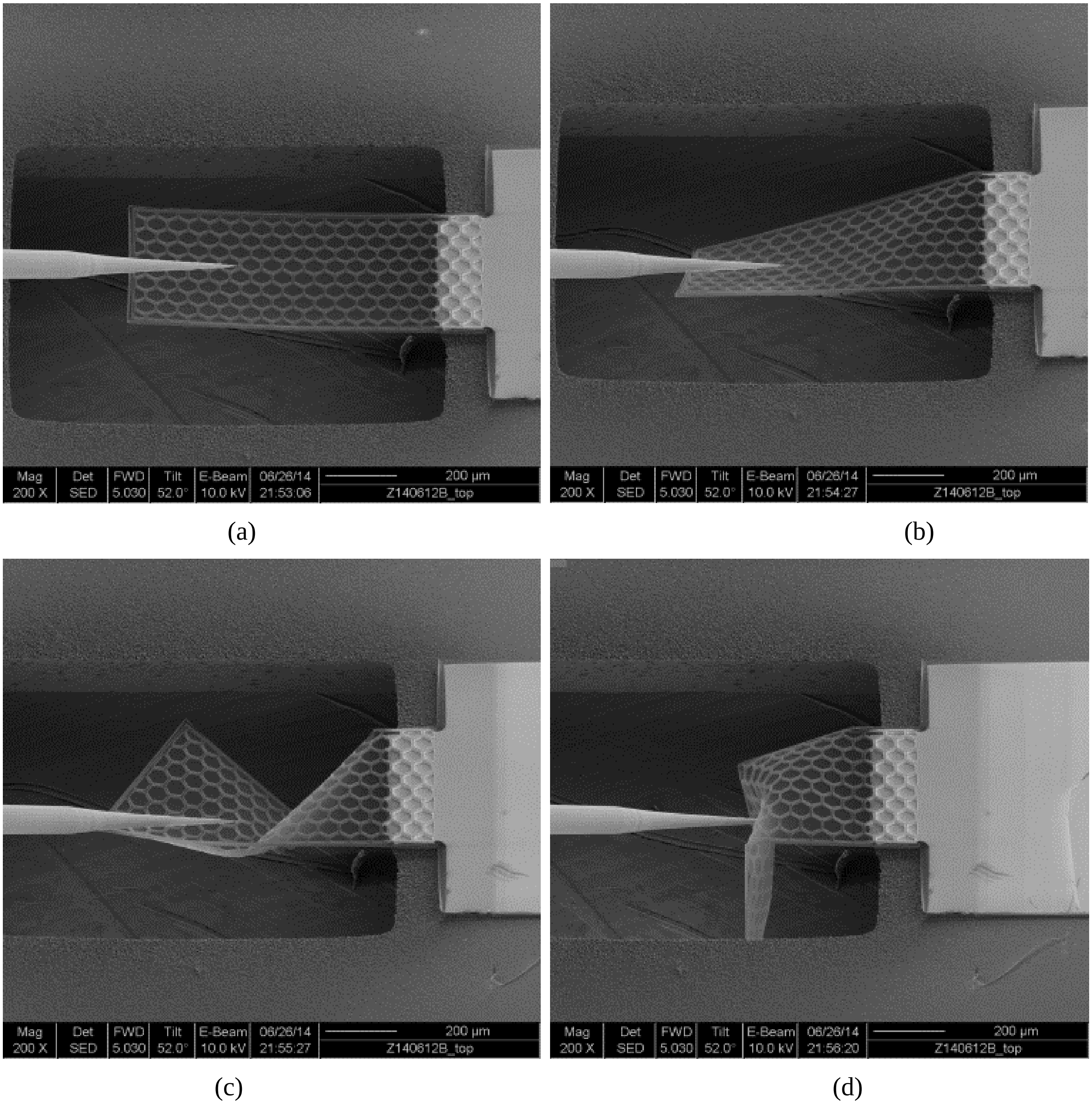

Supplementary Figure 3: *Sequential images of a structure with the ALD layer thickness of ~25 nm inside an FIB while being manipulated using a micromanipulator. The scale bar in all the pictures is 200 µm. The cantilever recovered its original shape, as in panel (a), after the micromanipulator was removed (see Supplementary Movie 1).*

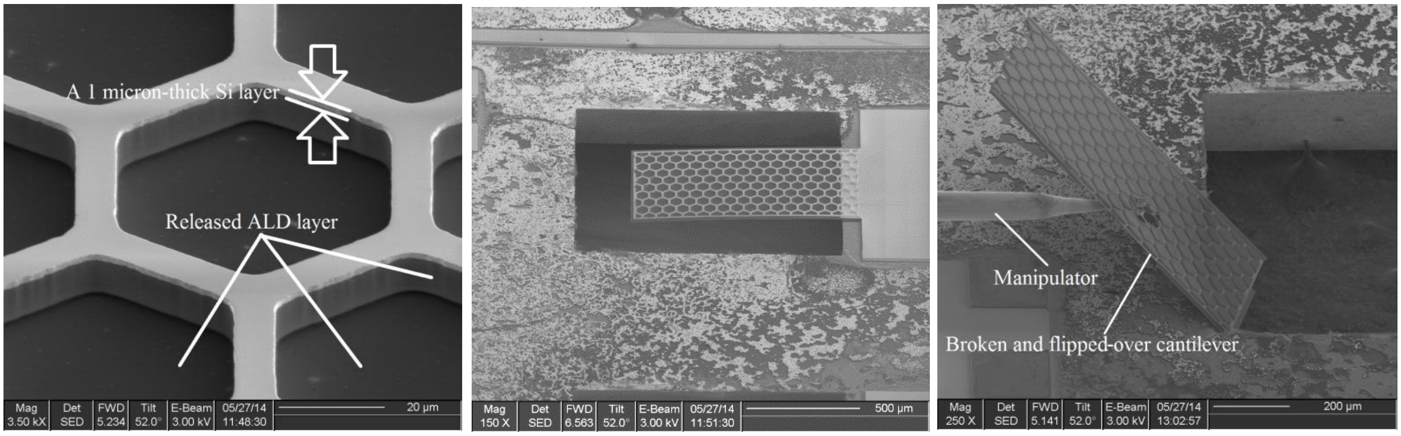

(a) (b) (c)

Supplementary Figure 4: *(a) A single cell of the cantilever showing the released and non-released thicknesses of the cantilever, (b) a cantilever with a 1 micron-thick layer of Si left, (c) the broken and flipped-over cantilever after manipulation.*

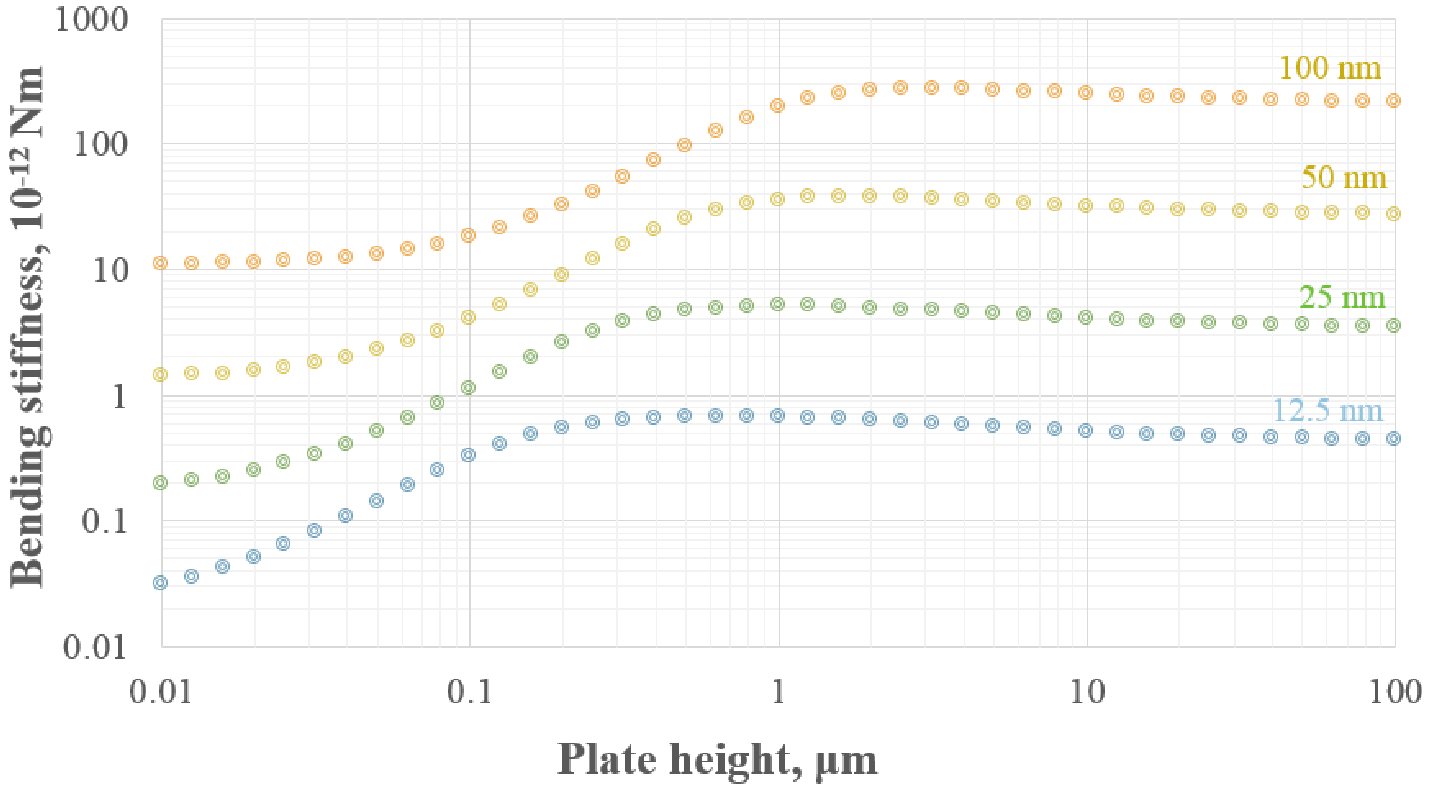

Supplementary Figure 5: *The simulated flexural rigidity as a function of cell height for hexagonal honeycombs with rib width w_r =10 µm, cell diameter width d_c = 50 µm, and film thickness t_f = 12.5, 25, 50 and 100 nm. The Young's modulus is assumed to be 130 GPa.*

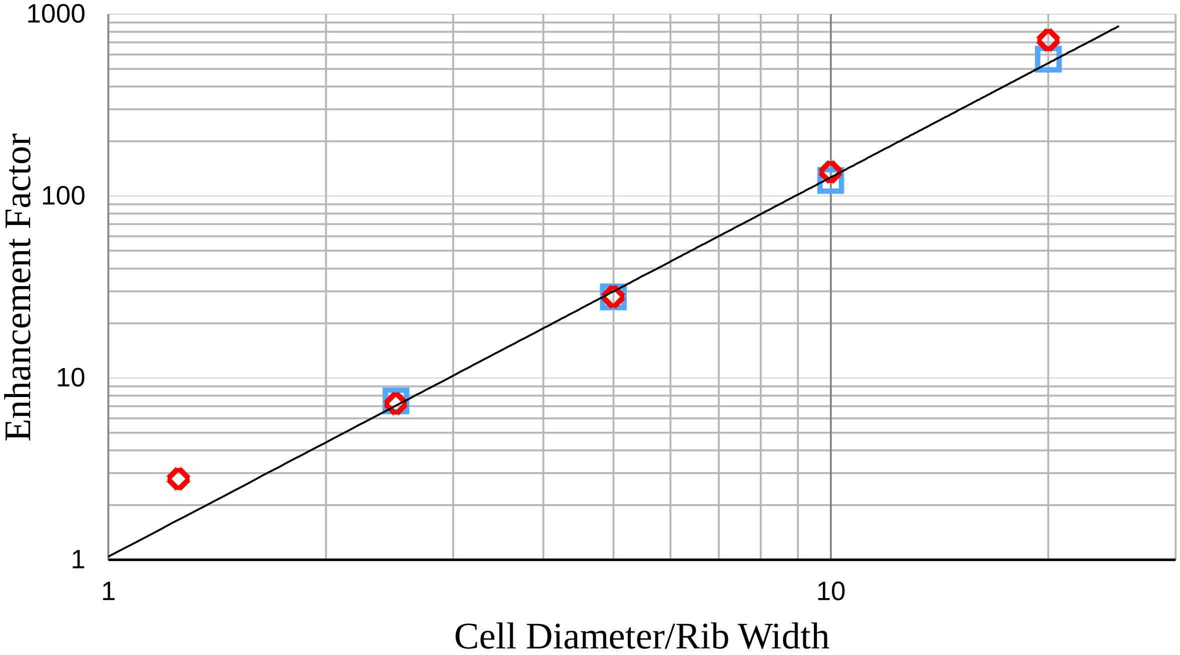

Supplementary Figure 6: *The enhancement factor as a function of the ratio of the cell diameter to the rib width. Red diamonds show results for a fixed cell diameter of 50 μm and varying rib widths (2.5,5,10,20, and 40 μm), while blue squares show results for a fixed rib width of 10 μm and varying cell diameter (25, 50, 100, and 200 μm). In both cases, the enhancement factor is reasonably well described by a simple formula* $EF \approx (\frac{d_c}{w_r} + 1)^2$ *(solid black line).*

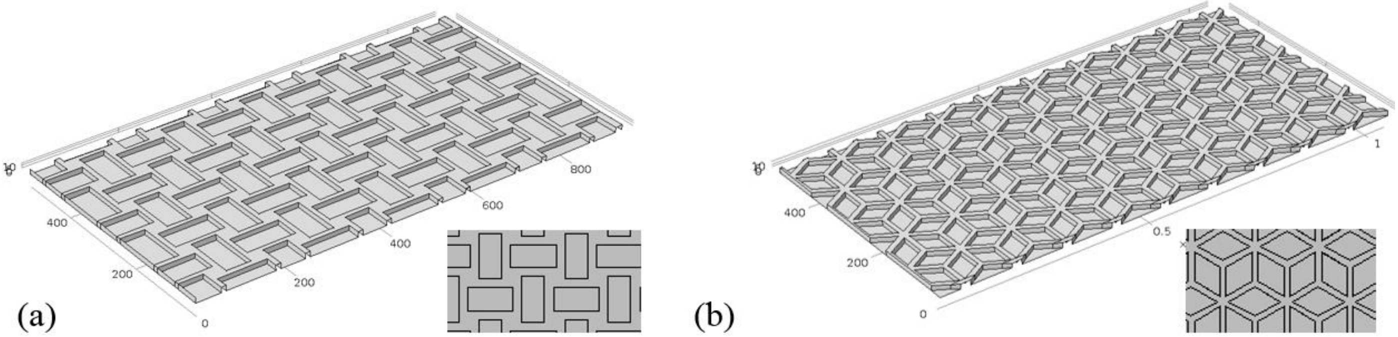

Supplementary Figure 7: *Alternative geometries of plate metamaterials that possess the property that every plane perpendicular to the plate must intersect vertical walls of the structure: basketweave (a) and rhombille (b). The insets show top-down views of both geometries.*

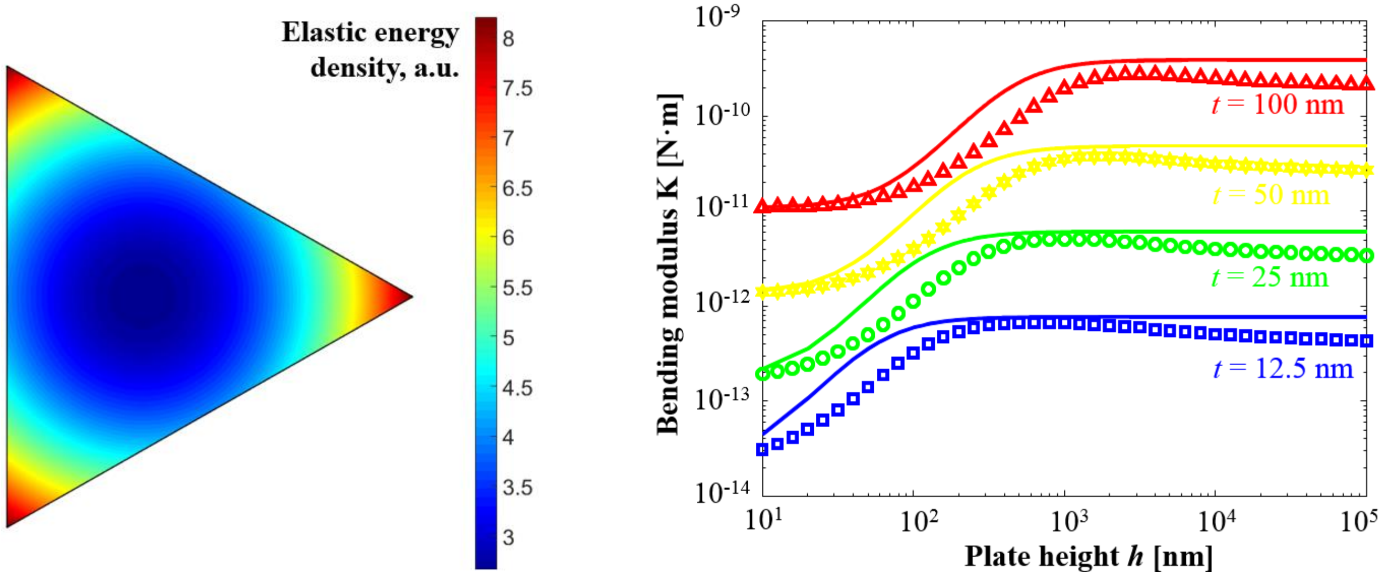

(a) (b)

Supplementary Figure 8: *(a) Distribution of bending elastic energy (in arbitrary units) for a simply supported triangle with constant per-unit-length bending moments acting on all sides. Based on analytical expressions derived in Ref. [6]. (b) Bending modulus* K *as a function of plate geometrical parameters. We hold* $D = 50$ *microns,* $w = 10$ *microns fixed and plot the bending modulus as a function of height* h *for four different plate thicknesses. For each plate thickness the results from finite element simulations are plotted as symbols and the solid line represents the analytical derivation above. There is a good qualitative agreement between the two approaches in that for large values of* h *the bending modulus becomes a constant. The ratio of this large-*h *bending modulus and the flat-film stiffness* $K_2 = \dfrac{Et^3}{12(1-\nu^2)}$ *is the enhancement factor, EF.*

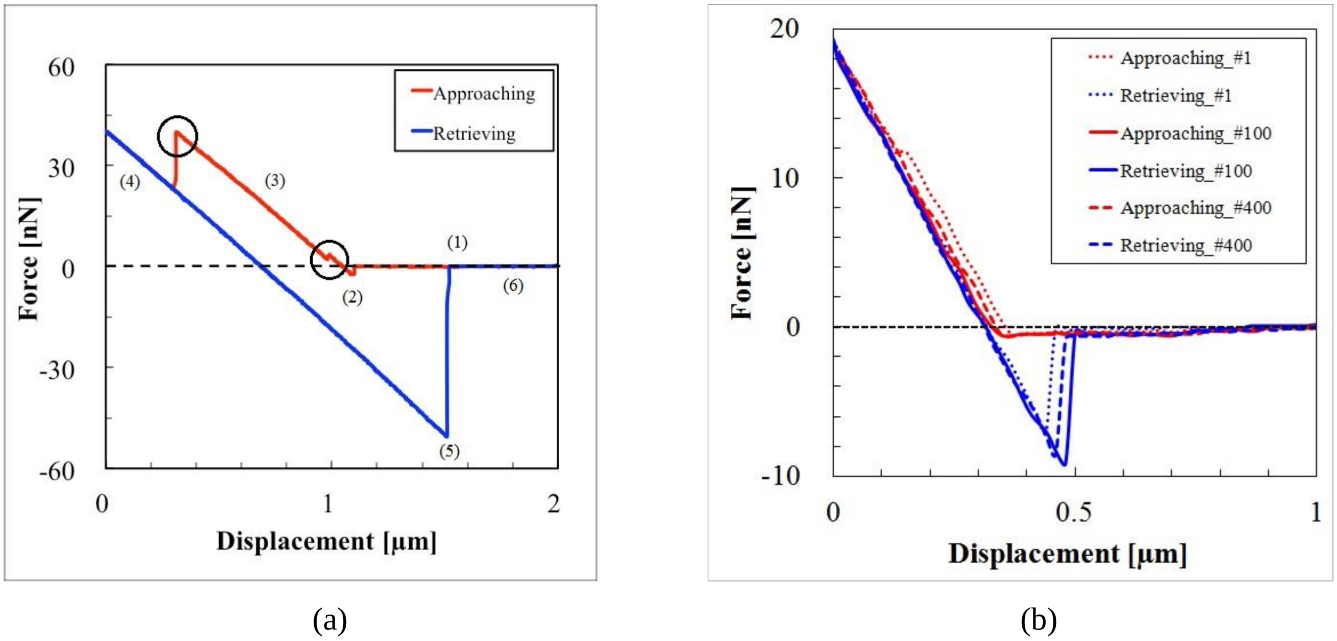

Supplementary Figure 9: *(a) a typical force versus displacement graph for a 10 um deep honeycomb structure with an ALD thickness of 72 nm. At point (1), as the AFM tip approaches the honeycomb cantilever, before making contact, the output force signal is zero. When the tip is close enough to the honeycomb cantilever (around a few tens of nanometers), the two snap into contact due to van der Waals forces (2). The honeycomb cantilever is pushed down more by the piezo drive (3) and the force increases until the maximum displacement is achieved and the AFM tip begins to retract (4). Before the tip completely separates from the cantilever, the force decreases significantly which is attributed to the adhesion force between the tip and the honeycomb cantilever (5). Finally, the AFM tip breaks free from the honeycomb cantilever when the elastic force of the AFM tip matures enough to dominate the adhesive forces (6). (b) Force-displacement curves of a cantilever after 1, 100 and 400 cycles.*

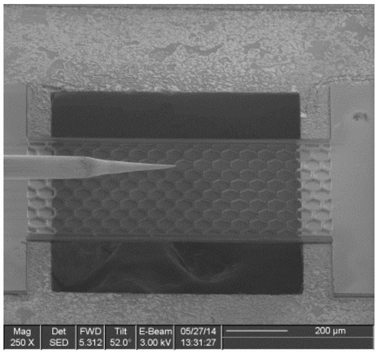

(a)

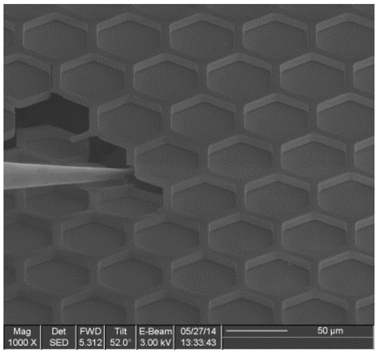

(b)

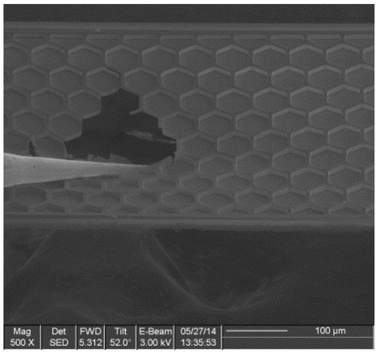

(c)

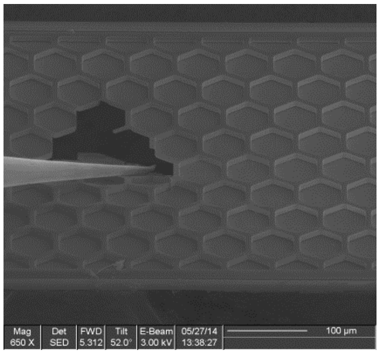

(d)

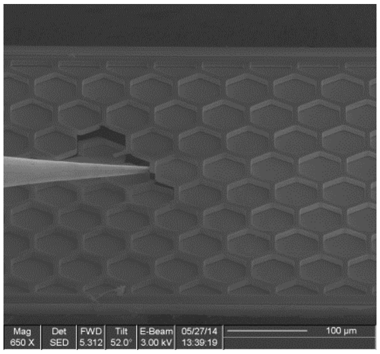

(e)

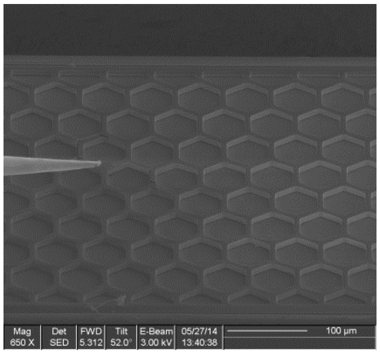

(f)

Supplementary Figure 10: *Shape recovery of broken cells. The scale bar is 200 in (a), 50 µm in (b), and 100 µm and other.*

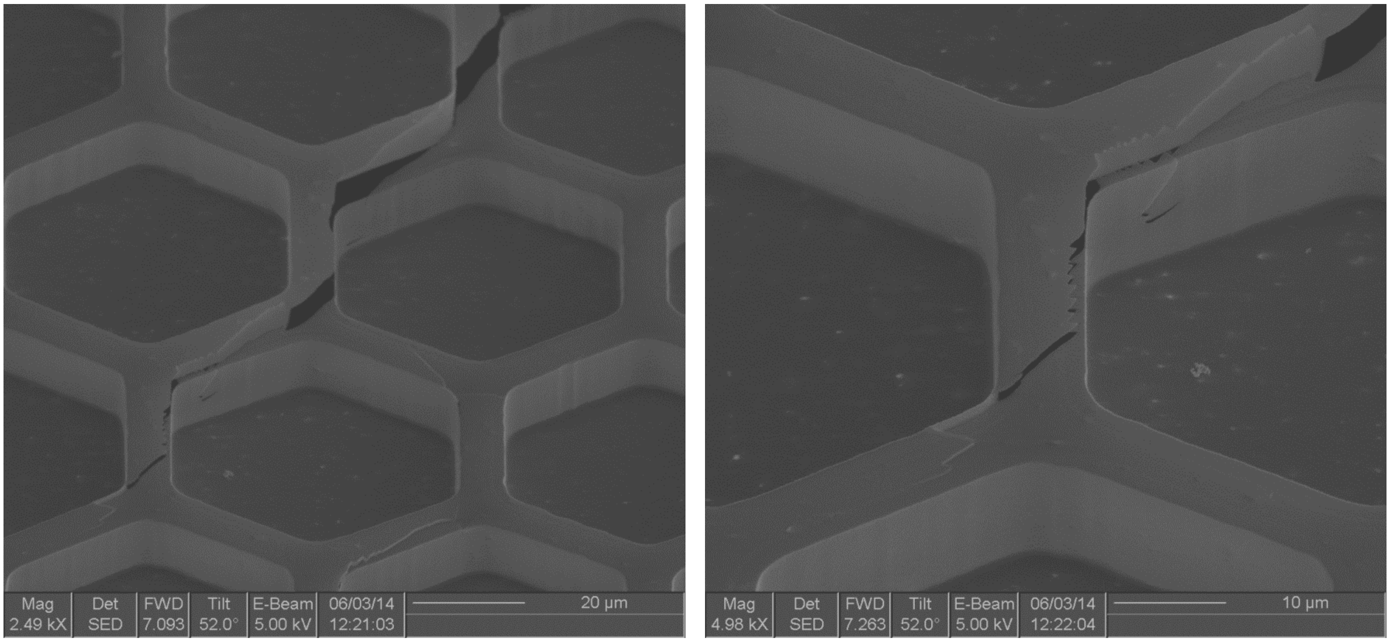

(a) (b)

Supplementary Figure 11: *Crack propagation and termination in a honeycomb ALD plate*

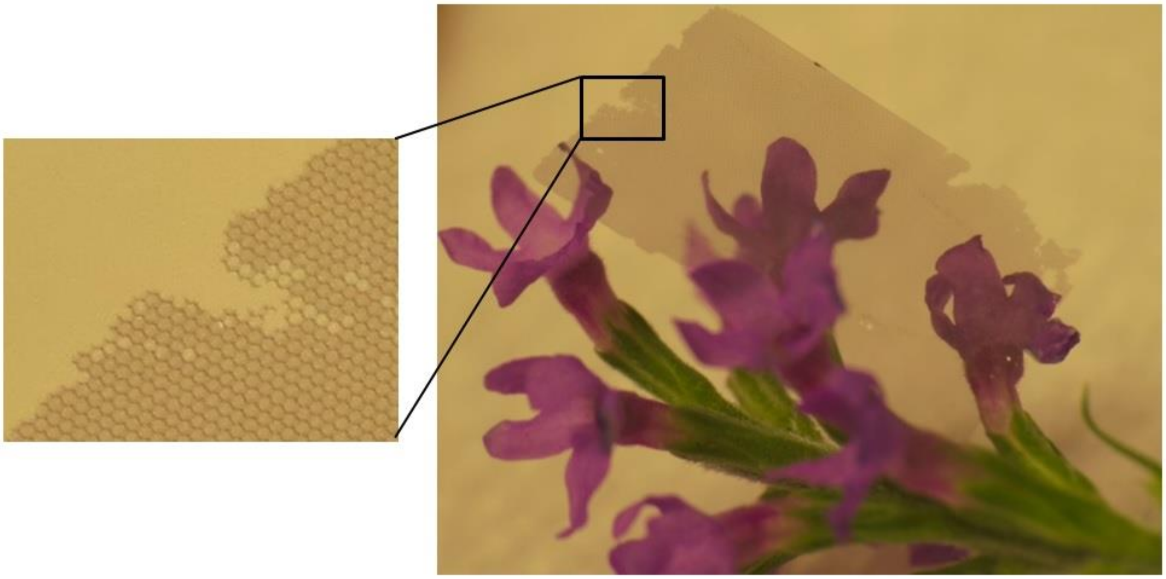

Supplementary Figure 12: *Defect localization of broken cells: A centimeter-long plate placed on flower petals but its edges are slightly damaged during the process. The inset zooms in on a section of the plate showing how the fraying of the structure's edges due to handling is limited because the cracks terminate after propagating a few cells and therefore do not affect the rest of the plate.*

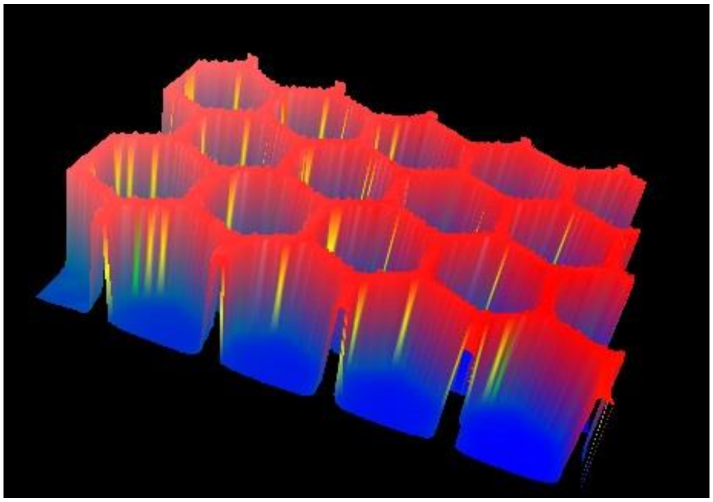

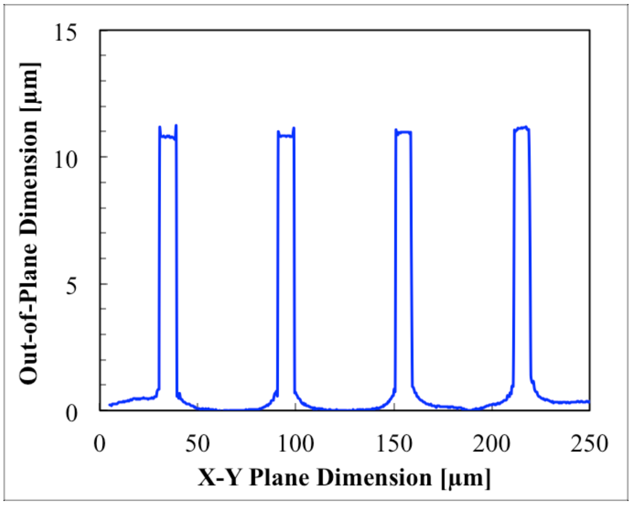

(a) (b)

Supplementary Figure 13: *(a) a surface topography image of the honeycomb structure with a height of ~11 µm. The dimensions in the Z direction are exaggerated for clarity. (b) The profile of a cross section of the structure.*

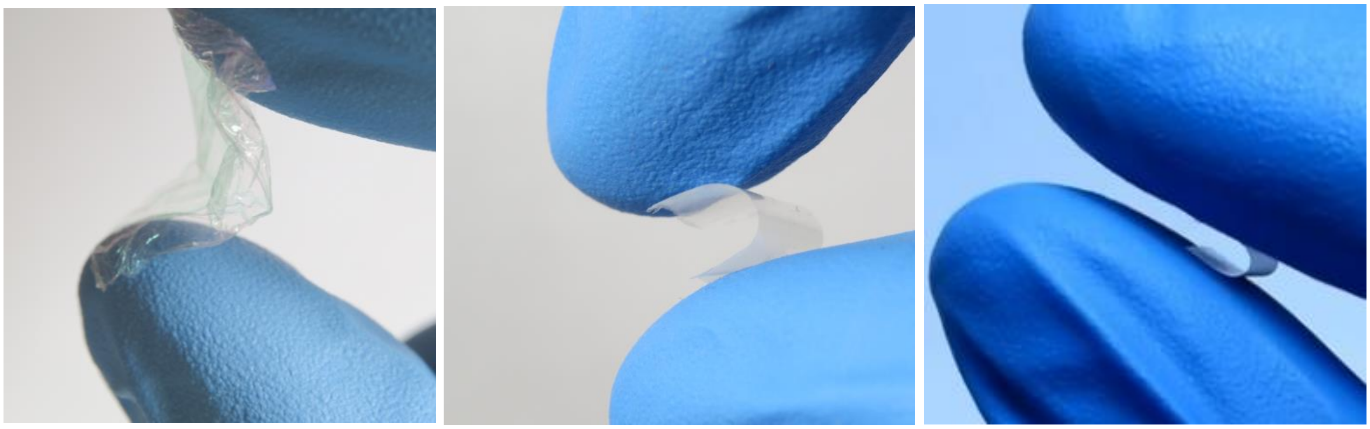

(a) (b) (c)

Supplementary Figure 14: *Macroscopic bending of (a) Mylar film with a thickness of 0.5 µm (a.k.a. "OS film"), (b) Mylar film with a thickness of 10 µm, and (c) ALD honeycomb plate with a 50 nm thickness.*

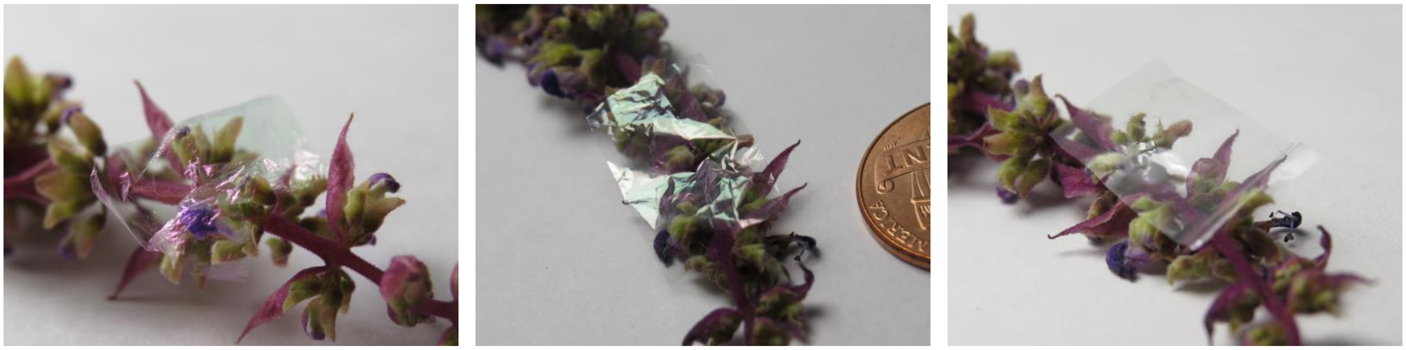

(a) (b) (c)

Supplementary Figure 15: *Sagging under self-loading due to gravity for Mylar films with a thickness of (a) 0.5 µm, (b) 2 µm, and (c) 5 µm. All films had lateral dimensions of 5 mm × 10 mm.*

Supplementary Table 1: *The flexural stiffness of different metamaterial plate patterns as calculated using finite element simulations (COMSOL). The characteristic cell size was fixed at 50 and 100 μm for the purposes of this comparison. The film thickness is 50 nm and the Young's modulus is 130 GPa.*

Characteristic cell dimension	Honeycomb	Basket weave with optimized rectangle width	Rhombille
50 μm	3.1×10^{-11} N·m	6.2×10^{-12} N·m	1.0×10^{-11} N·m
100 μm	1.6×10^{-10} N·m	7.2×10^{-12} N·m	2.0×10^{-11} N·m

Supplementary Note 1: Fabrication process & SEM images

Supplementary Figure 1a shows a die with all three types of millimeter-scale device fabricated out of plate metamaterial: cantilevers, doubly clamped beams, and rectangular plates clamped on all four sides, while Supplementary Figure 1b shows one centimeter-scale cantilever on wafer. The schematic of the fabrication procedure is outlined in Supplementary Figure 1c. The fabrication started with a double side polished Si wafer. SiN films with a thickness of 180 nm were deposited on both sides using PECVD. A hexagonal lattice of regular hexagons (honeycomb structure) with a depth of 10 and 14 µm were patterned on the front side of the silicon wafer using photolithography and reactive ion etching (RIE). The back side was patterned via photolithography and RIE etching of SiN to create openings for subsequent release. Next, the SiN mask was removed from the front side using RIE etching and the ALD layer was then deposited using trimethylaluminum (TMA) and water precursors. The deposition rate was measured using an ellipsometer to be 1.18 Å/cycle at 250° C.

In order to pattern the ALD layer, a thick layer of SPR 220 resist (MicroChem Corp.) was spin-coated on the structure. After the spin coating and soft baking at 105°C, the wafer was cooled down slowly to make sure the photoresist did not crack. After photolithography, inductively coupled plasma etching (ICP) with a BCl_3-based chemistry process was used to pattern the alumina ALD layer [1]. Most of the silicon under the ALD plate was removed using anisotropic KOH etching. Before placing the wafer in KOH, the top surface was covered with ProTEK B3 (Brewer Science Inc.) to prevent the ALD layer from being etched in the KOH solution. Also, since PECVD SiN does not cover the edge of the wafer, a protective chuck was used to keep the edge inaccessible. A silicon etching rate of 75 µm/hour was measured at 80° C in the 30% KOH solution. By accurately timing the KOH etching process, it was possible to stop the process ~ 20 µm from the top surface. The exact depth was measured using a Zygo white-light optical profilometer. After that, the ProTEK layer was removed and a 15-minute oxygen plasma was performed to remove any remaining polymer residue. Isotropic dry XeF_2 etching was used for the final release of the structure. Approximately 100 cycles (30 sec each) of XeF_2 etching with a ratio of 3.2:2 (XeF_2:N_2) was needed to completely release the structures.

Supplementary Figure 2a and 2b compare the scanning electron micrographs of a cantilever under different acceleration voltages. Under high acceleration voltages the structure is almost transparent in the SEM (Supplementary Figure 2a). The structure becomes more opaque when the acceleration voltage in the SEM is reduced to 2 kV (Supplementary Figure 2b).

Supplementary Note 2: Shape recovery after extreme deformation

In order to study the flexibility and shape recovery property of the structure, we used a micro-manipulator inside a focused ion beam (FIB) microscope. Honeycomb cantilevers with different ALD layer thicknesses underwent complex loading and deformation. The cantilever recovered its shape after each set of deformations without showing any damage. Structures with thinnest ALD layer films were able to withstand the highest level of deformation repeatedly without damage. Supplementary Figure 3 illustrates a set of sequential images of a cantilever with a thickness of 25 nm under different loading showing its flexibility and shape recovery. The corresponding video is also available as Supplementary Movie 1.

Due to relatively slow ALD deposition rates (~ 0.1 nm per cycle), we could not easily compare the flexibility and robustness of the ultrathin ALD plates (<100 nm) to those of much thicker ALD plates (~ 1µm). However, it was possible to contrast the behavior of the thinnest ALD plates to the behavior of various thicker structures that included some residual silicon. In general, such structures were much less flexible and broke easily. Supplementary Figure 4 shows a cantilever with a 1 micron-thick layer of Si left before and after it was broken. See also Supplementary Movie 1. While we attribute the observed breaking of the cantilevers with residual silicon primarily to high much larger thickness of the silicon layer, it may possibly be caused by other factors specific to two-layer Al2O3-Si composite samples, such as internal stress gradients due to a moduli mismatch.

Supplementary Note 3: Flexural stiffness of honeycomb plates and alternative geometries

In the limit of small linear deformations, the bending properties of a plate along a given direction is described by a bending stiffness $K = M/\kappa$, where M_{xx} is the bending moment flexing the plate along the given direction (x), and κ is the curvature of the plate along the same direction. (In general, the bending stiffness is a second-rank tensor, but this can be neglected for pure bending of plates with isotropic bending properties). For a plate with a uniform cross section along the bending direction, the bending stiffness is given simply by $K = EI/(1-\nu^2)$, where E is the Young's modulus of the underlying material, and I is the area moment of inertia of the plate's cross section per unit width of the plate. For a perfectly planar (unpatterned) film of thickness t, the bending stiffness is therefore given by the formula $K = Et^3/12(1-\nu^2)$.

When the plate consists of large number of cells (as in the case of both millimeter and centimeter plates we have fabricated), its small-deformation bending properties can also be described by an effective bending stiffness. For complex structures like the honeycomb plates described in this letter, there is no simple analytical formula relating the bending stiffness to the parameters of our cell structures (i.e., film thickness, rib width, cell height, and cell diameter). However, the effective bending stiffness can be inferred from the results of numerical simulations or experiments. This can be done either using the definition of the bending stiffness $K = M/\kappa$, i.e. by measuring the average curvature of the plate for a given applied moment, or indirectly by determining the spring constants of, e.g., cantilevers made from the same cellular plate metamaterial. In the limit of very long cantilever (Euler-Bernoulli approximation), the displacement due to shear deformations can be neglected and the spring constant by is simply given $k = 3W_{\text{cant}}K/L^3$ for a cantilever with a width W_{cant} and length L.

We have performed extensive COMSOL simulations of the bending stiffness of honeycomb plates, and found their bending properties isotropic within the accuracy of the simulations (typically, 10-20%). Supplementary Figure 5 shows how the bending stiffness changes with the changing height of the plate. At very heights ($h \ll t$), the hexagonal corrugation can be neglected and the stiffness saturates at the planar film value, $\approx \frac{Et^3}{12(1-\nu^2)}$. At high heights, the stiffness is also approximately constant, saturating for heights above ~1 μm. The enhancement factor, EF, is defined as the ratio of this saturated stiffness at high cell heights to the stiffness of a planar film. By correlating the results of multiple simulation runs, we have determined that the enhancement factor is almost independent of the film thickness t. For example in Supplementary Figure 5, the enhancement factor is approximately 25 for film thicknesses ranging from 12.5 nm to 100 nm, which covers the range of realistic ALD layer thicknesses. As a result, the enhancement factor is a function of only cell diameter, D, and rib width, w. Further numerical simulations revealed that the enhancement factor can be correlated to the ratio of these two parameters as follows: $EF \approx (D/w)^2$ (see Supplementary Figure 6). It is therefore clear that maximum bending stiffness is obtained by minimizing the rib width and maximizing the cell diameter. In practice, the rib width is limited by the errors of optical lithography, and in our experiments we have limited it to 10 μm for ease of fabrication. The diameter of the cell, in turn, should not be allowed to grow too large because large cell sizes reduce the robustness of the plates to crack propagation (see the corresponding section above). We have therefore limited the cell diameter to 50 μm.

In our experiments, we have focused on the honeycomb geometry due to its high symmetry and isotropic bending properties for small deformations. However, we have also considered a number of other lower-symmetry geometries using finite element simulations, e.g., basketweave-like geometry that has "cups" shaped as rectangles (Supplementary Figure 7a) and rhombille geometry that has "cups" shaped as rhombuses (Supplementary Figure 7b). Just like with honeycombs, the flexural stiffness of these cellular plates increased with decreasing rib width and increasing characteristic cell dimension (*i.e.*, the rectangle length for basketweave, the larger rhombus diagonal for rhombille, and the hexagon diameter for honeycomb). In the case of the basketweave pattern, we optimized the width of the rectangle numerically,

and the optimal width of the rectangle was typically approximately one half of its length. However, for a given characteristic cell size, both the rhombille geometry and the basketweave geometry with optimized width resulted in lower flexural stiffness than the honeycomb plates (Supplementary Table S1).

Supplementary Note 4: Theoretical analysis

While simulations give us some insight into the mechanical response of our patterned plates, additional insights into bending stiffness as a function of plate geometry can be obtained by analyzing the elastic energy stored in our plates using linear elasticity theory.

It is easiest to start with the honeycomb pattern in which hexagons of side $a = D/\sqrt{3}$ are separated by ribs of width w. Next to each hexagon vertex, there is an equilateral triangular region of side w, which is formed by the intersection of three ribs. Finite element simulations indicate that the stresses in this small triangular region are highly concentrated near each vertex, which is very similar to the stress concentration observed in simply supported triangle with constant per-unit-length bending moments acting on all sides [6]. We will use this insight in our calculation of an average bending modulus of the patterned plate. Consider a unit cell of area A of this honeycomb patterned plate. Let the area occupied by the small triangular regions per unit cell be A_2 and therefore $A_2 = A - A_1$ is the area of the remaining part of the unit cell. The corresponding matrices of bending and twisting moduli of these regions are K_1 and K_2, respectively. If a constant moment per unit length $\mathbf{M} = [m_x\ m_y\ m_z]$ is acting on the unit cell then the elastic energy stored in the patterned plate per unit cell can be written as $U = \frac{1}{2}A_1\mathbf{M}^T K_1^{-1}\mathbf{M} + \frac{1}{2}A_2\mathbf{M}^T K_2^{-1}\mathbf{M}$. Equating this energy to the expression for the whole plate $U = \frac{1}{2}A\mathbf{M}^T K_{\text{eff}}^{-1}\mathbf{M}$, we see that the average bending modulus K_{eff} for the patterned plate is given by $A\,K_{\text{eff}}^{-1} = A_1K_1^{-1} + A_2K_2^{-1}$. Now, $A = \frac{\sqrt{3}}{2}\left(\sqrt{3}a + w\right)^2 = \frac{\sqrt{3}}{2}(D + w)^2$ and $A_2 = \frac{\sqrt{3}}{2}w^2$. For large plate heights, the areas A_1 with vertical walls become very stiff, $K_1 \to \infty$, and the effective matrix of bending and twisting moduli is then given by $K_{\text{eff}} = \frac{A}{A_2}K_2$. Here, the ratio of areas $\frac{A}{A_2} = \left(\frac{D}{w} + 1\right)^2$ is the 'enhancement factor' in the bending stiffness of the plate. If we consider bending along one direction only then our finite element calculations agree very well with this formula for the enhancement factor if we take $K_2 = \frac{Et^3}{12(1-\upsilon^2)}$, where E is the Young's modulus of the plate material, ν is its Poisson ratio and t is the film thickness. The bending modulus K_1 does not enter the expression for the average bending modulus of the patterned plate in the limit of large plate heights. This is because most of the elastic energy resides in triangular areas A_2 due to stress concentration near the vertices of the hexagons (Supplementary Figure 8a).

However, for larger values of w or smaller values of height h, we need to account for K_1 to compute the average bending modulus of the plate. This can be done using the parallel axes theorem for computing moments of inertia. If the difference in height between the large hexagonal regions and the narrow ribs is h then the bending modulus $K_1 = \frac{E}{12(1-\upsilon^2)}\left[t^3 + 3th^2 + \frac{2th^2}{a+w}\right]$. Using this expression we computed the average bending modulus $K$of the plate as a function of D, h, w and t. The results are shown in Supplementary Figure 8b as lines of different colors for different plate thicknesses. The symbols are the result of finite element calculations. It is clear from this figure that our analytical approach captures the trend in the variation of the bending thickness quite well.

The same ideas as above can be applied also to plates with other patterns as long as we can assume that the bending moment per unit length is constant throughout the structure. In general, if there are n regions with different bending moduli in a unit cell of total area A, then the average bending modulus is given by $K^{-1} = \frac{1}{A}\sum_{j=1}^{n} A_j K_j^{-1}$, where A_j is the area of region j, K_j is its bending modulus, and $A = \sum_{j=1}^{n} A_j$. We can apply this idea to plates with rhombille patterns. With these patterns high stresses occur again in small regions at the vertices of each rhombus where (1) three channels meet (called 3-fold vertices), and (2) six channels meet (called 6-fold vertices). This geometry is visualized in Supplementary Figure 7b. If the side of each rhombus is a and the width of the ribs between them is w_r, then the area of a unit cell of this pattern is $A = 2\sqrt{3}\left(\frac{\sqrt{3}}{2}a + w\right)^2$. The area per unit cell occupied by the 3-fold vertices is $A_2 = \frac{\sqrt{3}}{2}w^2$ and that occupied by the 6-fold vertices is $A_3 = \frac{3\sqrt{3}}{2}w^2$. The bending moduli in both these regions are $K_2 = K_3 = \frac{Et^3}{12(1-\upsilon^2)}$. The rest of the unit cell has area $A_1 = A - A_2 - A_3$ and bending modulus K_1, which can be computed using the parallel axis theorem again. The trends of the bending modulus as a function of $a, h, w,$ and t for these plates are the same as those seen for the honeycomb patterns, but the bending modulus for the rhombille patterns is lower than those of the honeycomb patterns for the same values of the geometrical parameters.

Supplementary Note 5: Atomic force microscope (AFM) measurements

AFM measurements of force-displacement curves were performed using an Asylum atomic force microscope at room temperature and under ambient conditions. Two different types of AFM probes (NANOANDMORE, U.S.A) were used for the characterization. For the honeycomb cantilevers without the vertical sidewalls, the nominal spring constant of the AFM tip was ~0.01 N/m, and the length, width, and thickness of the cantilever were 125 µm, 34 µm, and 350 nm, respectively. The measurements on the honeycomb cantilevers with the vertical sidewalls were conducted using an AFM probe with a nominal spring constant of 2 N/m and a cantilever length of 225 µm, width of 27 µm, and thickness of 2.7 µm. To increase the reflectivity, the tip was coated with Al on the reflecting side. According to the manufacturer, the tip radius was less than 10 nm. Before the measurement of the ALD plates, the spring constant of the tip, K_{tip}, was obtained using a thermal noise method [2]. Prior studies have shown that this method has a measurement error of up to ~10% but is more accurate than many other alternatives [3].

After calculating the spring constant of the tip as well as obtaining the inverse optical lever sensitivity (InvOLS), a central load was applied at the middle of the free end of the cantilever using the tip. The load was in the range of 0.5 to 100 nN which is calculated by multiplying the trigger point (0.25V to 0.5V), InvOLS (~100 nm V^{-1}), and the spring constant of the cantilever (0.01 or ~2 N m^{-1}). The beam displacement can be calculated from $\delta_h = \Delta Z_p - \Delta Z_c$, where δ_h, ΔZ_p, and ΔZ_c are the honeycomb cantilever deflection, the AFM piezo travel distance, and the AFM cantilever deflection, respectively. Knowing that $1/K_{total}=1/K_{tip}+1/K_{beam}$, and K_{total} is the slope of the linear section of the force-displacement graph, the spring constant of the beam can be calculated.

One representative force-displacement graph is shown in Supplementary Figure 9a. In this experiment, the sharp AFM tip punctured the ALD film in two stages, as illustrated by the circles in Supplementary Figure 9a. Note that the slope of the force displacement curve remained constant before and after puncture events, meaning that the puncture events do not affect the measurement of the spring constant significantly. The adhesion force between the tip and the cantilever was relatively large at ~50 nN due to puncture of the film. This adhesion force is generally affected by the morphology of the surface of the sample, material of the tip and the cantilever, and the environment [4]. In experiments where no puncture signature was observed, the adhesion force was on the order of 10 nN, as can be seen from Supplementary Figure 9b.

Cyclic bending tests were performed to study the long-term stability of the mechanical properties of cantilevered honeycomb plates. A plate with a honeycomb depth of 10 um and an ALD layer thickness of 72

nm was bent and unloaded 400 times consecutively over the period of several hours. The spring constant of the beam did not change significantly throughout the process. Also, the beam was able to recover its initial shape after the bending. We did not observe any defects or a sign of failure. Supplementary Figure 9b illustrates the force-displacement (F-D) graphs after 1, 200, and 400 cycles. As can be seen here, there is no sudden drop in the applied forces and they are all superimposed with the same slope. This proves that no structural failure or plastic deformation happened in the cantilever during the experiment, and the beam maintained its original spring constant.

Supplementary Note 6: Failure mechanisms, defect localization, and shape recovery of broken cells

During some of the micromanipulator experiments, the plates were intentionally punctured by a shart probe, allowing us to explore some of the typical failure mechanisms of the plate metamaterials. The cracks appeared predominantly on the top surface of plate, which contains the areas of stress concentration near the vertices of triangles/hexagons as discussed above. The microscale cellular structure of our plates increases their robustness because any crack has a high probability of terminating at the vertical walls. If a crack happens due to the fabrication defects or external damage by a tip, it typically does not propagate more than a few cells. Also, after unloading, the broken cells return very close to their original position, so that the damage is not easily noticeable. An example can be seen in Supplementary Figure 10. The sharp tip of the manipulator penetrates the double clamped plate and breaks a few cells in the immediate vicinity (Supplementary Figures 10a-10c). As the manipulator is pulled back, the cells recover their original shape and the holes close up almost perfectly (Supplementary Figures 10d-10f).

As can be seen in Supplementary Figure 11a, a crack line typically does not advance through vertical walls and stays localized on the top surface. As a result, the crack cannot propagate in a straight line and has to deflect many times. This increases the total fracture surface area and energy absorption, making it likely that the crack will stop at one of the bends (Supplementary Figure S11b). The same defect localization mechanisms manifest themselves in macro-scale plates as well, as illustrated in Supplementary Figure 12. It is therefore important to keep the cell size of plate metamaterials reasonably small, providing many opportunities for defect localization, which dramatically increases the robustness of the plate metamaterial.

Supplementary Note 7: Profilometry

Supplementary Figures 13a and 13b illustrate a 3D image of the topography of the honeycomb structure as well as its cross-sectional analysis, respectively, obtained using a Zygo white light optical profilometer. The side walls were not completely vertical due to the depletion of the fluorine spices at the bottom of the honeycomb structure and slow transport of the etching products out of the trenches [5]. Depending on the depth of the etching, tapering angles between 5 to 15 degrees were observed in different structures. Also, side wall etching causes the width of the ribs to be slightly different in samples with different depths, and relative to the design and simulation dimensions.

Supplementary Note 8: Comparison of a free-standing honeycom ALD plate with thin polymer sheets

The thinnest plastic film that is commonly available is 0.5-μm-thick Mylar film, also known as “OS film”. These films have an areal density of ~0.7 g m^{-2}, compared to as little as ~0.1 g m^{-2} for our honeycomb plates. In addition, the Mylar film has very different bending properties as illustrated in Supplementary Figure 14a. The plastic film wrinkles very easily, sticks easily to itself or nitrile gloves, and does not recover the original flat shape when released from the fingers. In fact even thicker films of Mylar that we tested (1μm, 2 μm) suffered from the same inability to recover the original shape after direct handling with gloved hands. Only films with a thickness of ~10 μm or higher maintained their shape as well as our ALD plates (compare Supplementary Figures 14b and 14c).

In order to characterize the ability of plates to maintain their own shape under gravity, it is useful to consider the figure of merit that is the ratio of bending stiffness to areal density. Plates of similar size and with similar figures of merit should bend under their own weight in a similar way. Given that our honeycomb

plates made of 50-nm-thick alumina have an areal density of 0.25 g $m^{-2} = 2.5 \times 10^{-4}$ kg m^{-2} and a bending stiffness of 2.4×10^{-11} N m, the corresponding ratio is approximately 2.4×10^{-11} N m/2.5×10^{-4} kg $m^{-2} \approx 10^{-7}$ m^4 s^{-2}. For planar sheets (e.g. Mylar sheets), the bending stiffness is simply given by $\frac{Et^3}{12(1-\upsilon^2)} \approx \frac{Et^3}{12}$ and the areal density by ρt, so their ratio is $\approx \frac{Et^2}{12\rho}$. Assuming a Young's modulus of 1 GPa and density of 1400 kg/m^3 for Mylar, it is easy to calculate that a Mylar sheet with a film thickness of 1.6 μm would have the same figure of merit as our honeycomb plates. In practice, Mylar films with a thickness of 2 μm still exhibited some tendency to sag around the edges due to gravity. Only films 5 μm or thicker did not sag noticeably and therefore maintained their shape as well as honeycomb plates (Supplementary Figure S14). However, even such thicker Mylar films were typically not as flat as our ALD plates (compare Supplementary Figure 15 to Supplementary Figure 12 above or Fig. 3c in main text) due to prior plastic deformations that are almost unavoidably introduced when handling Mylar films.

Supplementary References:

1. Yang, X., Woo, J. C., Um, D. S., Kim, C. Dry etching of Al2O3 thin films in O2/BCl3/ Ar inductively coupled plasma. *Trans. on Electr. Electron Mater*. **11,** 202-205 (2010).
2. Heim, L., Kappl, M., Butt, H. Tilt of atomic force microscope cantilevers: effect on spring constant and adhesion measurements**.** *Langmuir* **20,** 2760-2764 (2004).
3. Butt, H., Jaschke, M. Calculation of thermal noise in atomic force microscopy. *Nanotechnol*. **6,** 1-7 (1995).
4. Feng, X., Keiviet, B., Song, J., Schon, P., Vancso, G. Adhesion forces in AFM of redox responsive polymer grafts: Effects of tip hydrophilicity. *Applied Surface Science*, **292,** 107-110 (2014).
5. Wu, B., Kumar, A., Pamarthy, S. High aspect ratio silicon etch: A review. *J. Appl. Phys.* **108,** 051101 (2010).
6. Timoshenko S. P. and Woinowsky-Krieger S., *Theory of plates and shells*, 2nd edition, (McGraw-Hill, 1959).
